# The Acquired Ilio-Femoral Articulation

**Published:** 1889-01

**Authors:** 


### THE ACQUIRED ILIO-FEMORAL ARTICULATION.

Mr. W. Aburthnot Lane writes (in *Medico-Chirurgical Transactions, Vol. 53,*) of an undescribed method by which the superjacent weight of the body is transmitted in united or ununited fracture of the femur through an acquired ilio-femoral articulation and the bearing of the principle involved upon the surgery of the hip-joint.

In 320 subjects that he has examined he found several instances of fracture of the neck of the femur, in all of which but three union was complete. He was struck by the fact that in three out of the small number of these fractures, a very considerable portion of the superjacent weight of the body was transmitted to the femur by means of a very strong and dense process of bone, which projected from the front of the upper end of the femur, and articulated above by a flattened upper extremity with a corresponding quadrilateral depression situated immediately below the anterior inferior spinous process of the ilium, and encroaching to a varying extent upon this process. The opposing surfaces of bone, as well as the portions of bone immediately adjacent, were intimately united to one another by dense ligamentous tissue. The amount of pressure transmitted through this newly-formed articulation varied considerably in the three cases. In that in which the fragments had not united by bone, almost, if not the whole of the superjacent weight was transmitted through the acquired joint. In the two cases in which the fragments had united by bone the amount of weight transmitted by the head of the femur was considerable, in one case equaling, and in the other possibly exceeding that borne by the accessory articulation.

Mr. Lane directs attention to the immense mechanical advantages which the ilio-femoral articulation affords in cases of fracture of the femur in which there is much downward displacement of the head of that bone, and these advantages are especially obvious when the fragments move freely upon each other. He also calls attention to the manner in which this newly-developed articulation serves to diminish as much as possible the shortening of the limb both apparently and practically, to render it very much more useful than it would be without the pressure of such a powerful joint, and to reduce to a very considerable extent the deformity of the trunk, and particularly the extreme lordosis and unsteady gait that would otherwise ensue on the injury. He believes that the application of the knowledge of this mechanism will enable us to make very considerable strides in the operative surgery of the hip-joint.

Take, for example, the operation of excision of the hip-joint. The most successful operation gives comparatively unsatisfactory results. Apart from the shortening, the chief reason of the comparative failure of this operation seems to be, that the divided upper extremity of the femur is left in apposition with the outer aspect of the ilium, to make for itself a most insecure and unsatisfactory connection with that bone, in a position in or behind the original vertical plane of the acetabulum. This new articulation, on the security of which the usefulness of the limb depends, allows of a gliding movement between the bones, the upper extremity of the femur rising vertically to a variable extent when the weight of the body is thrown upon the excised limb.

From an examination of the mechanism of this ilio-femoral articulation, developed in the case of ununited fracture of the femoral neck, it is obvious that if in the operation of excision of the hip-joint the upper extremity of the femur were displaced upwards, forwards, and inwards, and were attached or fixed in position to the anterior inferior spinous process and to the bone immediately beneath it, we should have formed an ilio-femoral aaticulation that would differ in no way from, and would serve its purpose quite as well, as did that described. The mechanical advantages gained by the formation of such an ilio-femoral joint in cases of excision are enormous. Instead of the usually very feeble joint that, when unsupported, cannot, except in very rare cases, support the weight of the body, we would have an articulation that would sustain nearly as much strain as the original, which would be accompanied by much less deformity of the trunk both in standing and walking; and the deficiency in the length of the leg could be readily compensated without increasing the upward displacement of the femur and diminishing still further the usefulness of the joint in connection with that bone.

